# Case series of mycosis fungoides with clinical features suggestive of seborrheic dermatitis

**DOI:** 10.1016/j.jdcr.2026.06.033

**Published:** 2026-06-19

**Authors:** Alexa C. Kwang, Kacie R. Carlson, Christine J. Ko, Michael Girardi

**Affiliations:** a Yale School of Medicine, New Haven, Connecticut; b Department of Dermatology, Yale School of Medicine, New Haven, Connecticut

**Keywords:** cutaneous T-cell lymphoma, mycosis fungoides, seborrheic dermatitis

## Introduction

Mycosis fungoides (MF) is the most common subtype of cutaneous T-cell lymphoma.^1^ The clinical presentation can range from patches and plaques in early-stage (≤IIA) disease to thicker plaques, tumors, erythroderma, and blood/lymph node involvement in more advanced stages (≥IIB).^1^ There are also many clinical and pathologic variants of MF, including hypopigmented and folliculotropic mycosis fungoides (FMF).^1^ Due to this heterogeneity, MF is known to mimic various benign inflammatory dermatoses.^2^, ^3^, ^4^ This phenomenon contributes to diagnostic delays in early-stage MF, which increases the risk of inappropriate treatment and/or disease progression.^5^ It is therefore crucial to understand the similarities and differences in clinicopathologic features between MF and its mimics. Atopic dermatitis and psoriasis are 2 such conditions that may be misdiagnosed in patients with MF.^2^^,^^3^^,^^5^ Much less well described, however, is MF mimicking seborrheic dermatitis (SD), a chronic inflammatory condition characterized by erythema and scaling in a seborrheic distribution.^6^ Here, we present a retrospective case series of 3 patients with MF that presented with new lesions that showed clinical and/or early histopathologic features resembling SD.

## Case series

All 3 patients had prior diagnoses of MF and were being treated at Yale Dermatology. Patient demographic, clinical, histopathologic, and molecular data were ascertained from the electronic medical record. Patient 1 was an 84-year-old female with well-controlled MF on narrowband ultraviolet B (NB-UVB) therapy who presented with a scaly, erythematous patch in the right nasolabial fold (Fig 1, *A*). The lesion was clinically diagnosed and treated as SD. Six months later, the patient returned with a hyperkeratotic plaque with thick scale that was refractory to SD therapies ketoconazole and hydrocortisone 2.5% creams and subsequently to clobetasol cream (Fig 1, *B*). The clinical differential diagnosis included exuberant SD, squamous cell carcinoma in situ, and MF. Shave biopsy histopathology revealed epidermal hyperplasia with an atypical lymphoid infiltrate composed of CD3+ CD4+ epidermotropic and folliculotropic T cells consistent with MF (Fig 1, *C*). The lesion resolved with focal electron beam radiotherapy and additional oral bexarotene 150 mg for more generalized disease control. Despite this combination, the patient manifested MF activity elsewhere, including erythematous patches and plaques on one of the eyelids and bilateral extremities including palms and soles, with an ulcerated plaque on the third digit. Of note, epidermotropism and folliculotropism were similarly present in the eyelid lesion, but follicular involvement could not be evaluated in the shave biopsy of the sole due to absence of follicles on sections.

**Fig 1:**
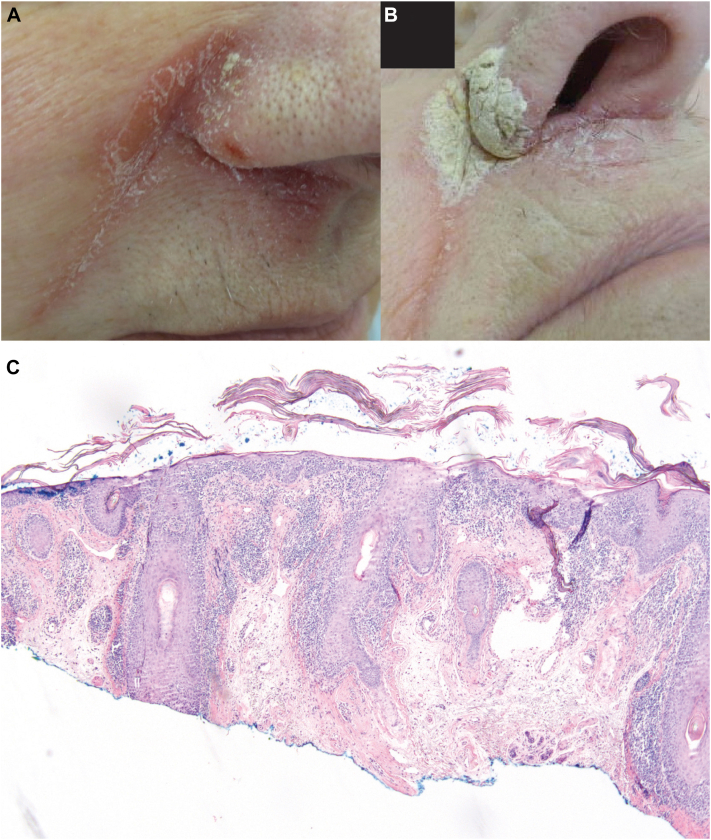
Clinical and histopathologic images of lesion progression in Patient 1. **(A)** Scaly, erythematous patch in the right nasolabial fold at initial presentation. **(B)** Hyperkeratotic pink plaque in the right nasolabial fold at 6-month follow-up visit. **(C)** Epidermal hyperplasia with moderately dense perivascular lymphocytes with exocytosis into the epidermis and hair follicle epithelium (hematoxylin-eosin, 40×).

Patient 2 was a 74-year-old female receiving NB-UVB for MF patch lesions on the palms, soles, and right posterior thigh. She had a history of scalp SD but manifested a new erythematous, pearlescent plaque in the right alar crease (Fig 2, *A*). Clinical evaluation prompted a differential diagnosis of SD, basal cell carcinoma, and MF. Shave biopsy revealed focal parakeratosis and nonspecific inflammation, and SD remained in the microscopic differential diagnosis (Fig 2, *B*). Seven months later, the patient returned due to lesion progression despite treatment with topical hydrocortisone 2.5% cream and tacrolimus 0.1% ointment, and oral bexarotene 75 mg prescribed for more general MF activity. Repeat shave biopsy showed a dermal and epidermal infiltration of CD3+ CD4+ atypical T cells consistent with tumor-stage MF (Fig 2, *C*). Bexarotene dose was increased to 225 mg daily, leading to partial reduction of the lesion (Fig 2, *D*). After ∼4 months, the lesion resolved with focal radiotherapy.

**Fig 2:**
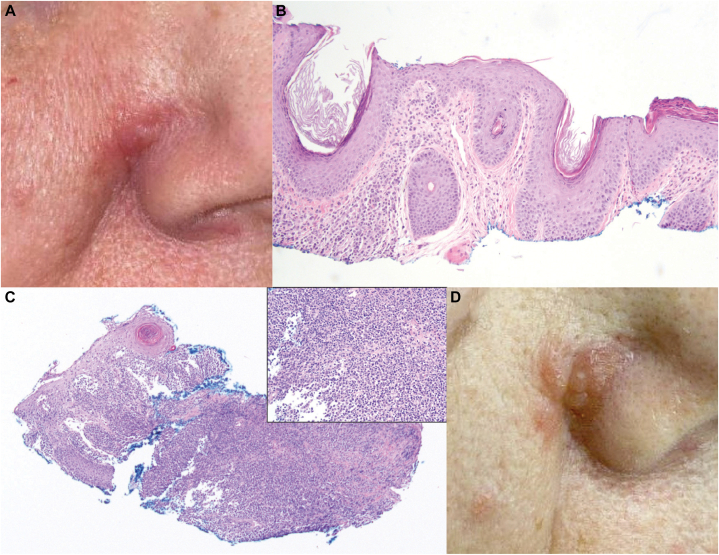
Clinical and histopathologic images of lesion progression in Patient 2. **(A)** Erythematous, pearlescent plaque in the right alar crease at initial presentation. **(B)** Mild parakeratosis, slight acanthosis, and dermal lymphocytic infiltrate (hematoxylin-eosin, 100×). **(C)** Dense lymphocytic infiltrate in the dermis with focal exocytosis at 7-month follow-up visit (hematoxylin-eosin, 40×; inset, 200×). **(D)** Pearly papules in the right alar crease after 1 month of treatment for lesion progression.

Over the course of her disease, Patient 2 experienced disease progression with additional disease activity on the eyelid, palms, soles, and extremities. One year after the resolution of the alar crease lesion, she was initiated on a clinical trial (NCT05079282, ONO-4685, anti-PD-1/CD3 bispecific antibody) for relapsed or refractory T-cell lymphomas including cutaneous T-cell lymphoma. After receiving the first dose, however, the patient experienced a substantial asymmetric worsening of patches and plaques with erosions, crusting, and purulent drainage on the temples, eyelid, cheeks, and nasolabial/perioral areas, that is, within the typical distribution of SD (Fig 3). A new lesion arose concurrently on the right arm, and shave biopsy confirmed progression of MF.

**Fig 3:**
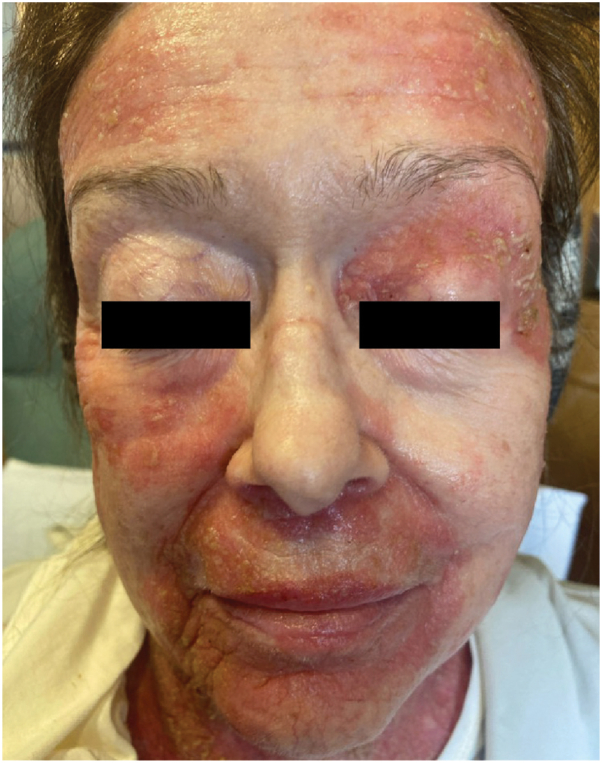
Asymmetric involvement of MF lesions on the face of Patient 2, including the bilateral temples, left eyelid, right > left cheek, and nasolabial folds. *MF*, Mycosis fungoides.

Patient 3 was a 70-year-old male with a history of eyebrow SD and patch/plaque-type of MF on NB-UVB who presented with a scaly, erythematous patch on the right medial upper eyelid at the inferior border of the eyebrow (Fig 4, *A*). The initial differential diagnosis included SD and MF. Shave biopsy showed histopathologic findings of spongiosis, compatible with an eczematous process including SD (Fig 4, *B*); however, a denser lymphoid infiltrate than typical for an inflammatory dermatosis alone prompted polymerase chain reaction for T-cell receptor (TCR) clonality that was positive for a clonal rearrangement. Together, the histologic and molecular assessments were consistent with MF. The eyelid lesion fully resolved with a regimen of hydrocortisone 2.5% cream 3 times a week and eyelid exposure during NB-UVB therapy. Over the disease course, the patient manifested other MF lesions including scaly erythematous patches on the dorsal hand, upper thighs/inguinal region, and buttocks.

**Fig 4:**
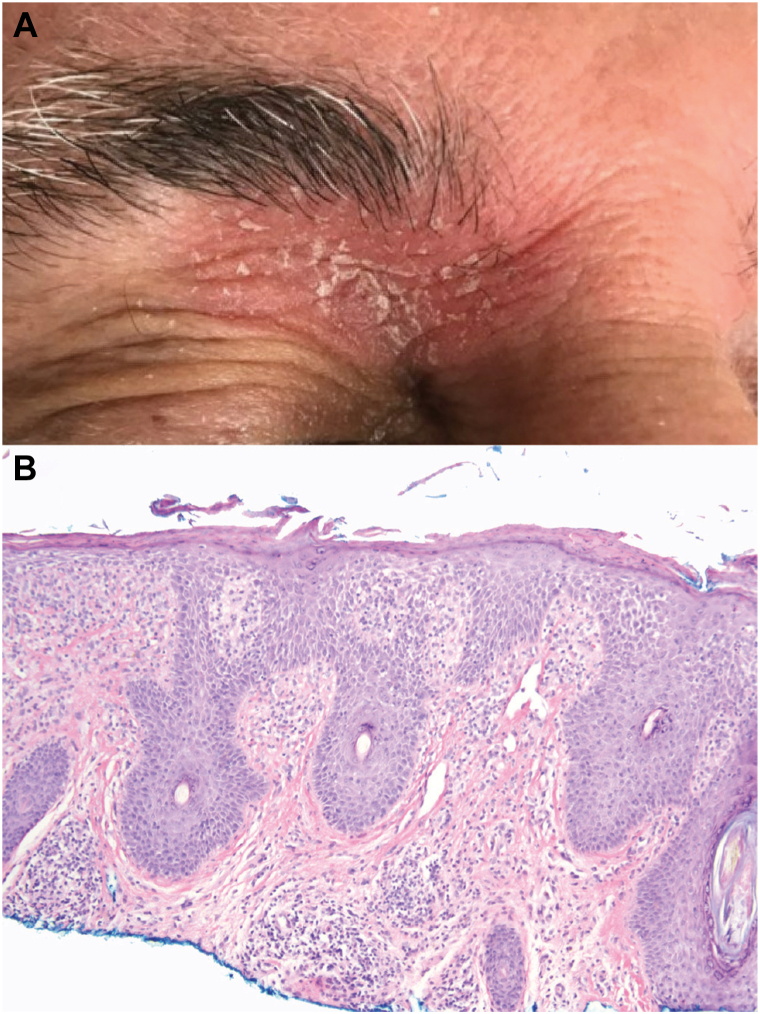
Clinical and histopathologic images of Patient 3 at initial presentation. **(A)** Scaly, erythematous patch limited to the right medial upper eyelid at the inferior border of the eyebrow. **(B)** Spongiosis with exocytosis below parakeratosis with a moderately dense perivascular lymphocytic infiltrate (hematoxylin-eosin, 100×).

## Discussion

This case series supplements the concept of MF as a mimicker of benign, immune-mediated dermatoses by describing SD-like MF, a distinct yet less well-characterized clinical manifestation of MF where diagnosis may be delayed. Since all 3 patients had prior history of MF, MF was appropriately considered in the differential diagnosis for the presenting nasolabial fold, alar crease, and eyelid lesions. However, factors like well-controlled MF activity at the time (Patient 1), concurrent history of SD (Patients 2 and 3), and lesion morphology (all 3 patients) raised suspicion for SD as well. In Patient 1, a single biopsy with histopathology was sufficient to clarify the clinically ambiguous presentation. Patients 2 and 3, in comparison, required repeat biopsies and polymerase chain reaction for TCR clonality, respectively, to definitively confirm the diagnosis of MF. With regard to Patient 3, we should note that chronic benign dermatoses (eg, lichen planus) can also exhibit low-level clonality.^1^ Although SD does not typically demonstrate T-cell clonality, false-positive TCR molecular studies may still occur, especially in cases of limited T-cell infiltrate.^7^ In our patient, however, the combination of clinical presentation, histopathology showing a denser-than-typical CD4+ T-cell infiltrate, positive clonality, and history of MF all strongly supported the final diagnosis of MF.

We considered several overarching pathomechanistic drivers of the SD-like MF variants evident in these cases. SD is a common dermatosis with an estimated global prevalence of 5%.^6^ It is thus conceivable that patients with a predilection for SD might provide a *locus minoris resistentiae* (ie, area of decreased resistance)^8^ for MF T cell skin-homing and lesion development within an SD distribution. Since the inflammatory milieu of chronic skin disease has been established as a driver of malignant cell transformation and progression in other lymphomas through local immunoregulatory and growth factor influences,^9^ pre-existing SD might foster a tumor microenvironment that promotes MF lesional progression and contributes to the variable treatment resistance observed in these cases. Consistent with this, an *in vivo* model of murine T-cell lymphoma previously demonstrated cutaneous tumor formation restricted to sites of 2,4-dinitrofluorobenzene‑induced inflammation.^10^

More specifically, given that the surface free fatty acid and barrier function alteration features of seborrheic skin support the colonization of *Malassezia* species that are known to activate antigen-presenting cells, that is, via Toll-like receptor-2, nucleotide-binding oligomerization domain-like receptors, and C-type lectin receptors,^11^^,^^12^ the resultant cytokine milieu may provide ongoing co-stimulation and survival signals for skin-homing malignant T cells and further support an MF-permissive tumor microenvironment. It is thus reasonable to consider whether the lesions initially diagnosed as SD in Patients 1 and 2 were truly SD at the time, predisposing the patients to the development, and subsequent lesional progression, of MF in the same locations. Nonetheless, there are key features shared between all 3 cases that argue for the lesions being MF from the outset rather than SD that enabled MF. Foremost, the unilateral, asymmetric lesions contrast with the generally bilateral, symmetric distribution of SD.^6^ Moreover, the plaque-like, progressive, and refractory nature of the lesions when treated with SD therapies, as well as the patients’ history/concurrence of MF, suggest that these cases were primarily MF with clinical features of SD.

The disease progression observed in Patient 2 after receiving the clinical trial agent was especially striking due to the specific distribution of patches/plaques on the temples, eyelid, cheeks, and nasolabial/perioral areas. This pattern overlaps with the typical facial presentation of SD in adults, for example, the forehead, eyebrows, eyelids, glabella, cheeks, and nasolabial folds.^6^ In addition to the described SD-like lesions, all 3 patients exhibited other sites of MF involvement, such as areas more commonly associated with classic MF, that is, acral areas, thighs/inguinal areas, and buttocks.^1^ Another notable parallel was the lesions on the eyelid, palms, and soles in Patients 1 and 2, suggesting an acral distribution predilection potentially similar to that of the palmaris et plantaris variant of MF.^13^ Patient 3 also showed MF involvement of the hand, albeit predominantly the dorsal aspect. It is possible that the persistence of the acral lesion affecting the nasolabial fold in Patient 1 was driven in part by folliculotropism, as FMF can impart an acral distribution and has been shown to exhibit resistance to first-line skin-directed therapies including NB-UVB.^14^ However, given the significant concurrent epidermotropism and the absence of characteristic FMF lesion morphologies (ie, follicular papules, alopecia, comedones, and/or follicular cysts),^1^ this appears less consistent with true FMF and may be more accurately described as MF with features of folliculotropism. Punch biopsies, particularly of the other lesions within the seborrheic and acral distribution like the sole of the foot, may have been helpful to formally evaluate for widespread and/or prominent follicular involvement.

Taken together, these 3 patients with classic MF developed new lesions that displayed clinical features of both SD and classic MF, further contributing to the spectrum of atypical presentations that can complicate MF diagnosis.^5^ Our cases demonstrate why new, morphologically ambiguous skin lesions in the setting of prior history of MF should necessitate a thorough evaluation for an underappreciated manifestation of MF. Other important clues that can help distinguish lesions of SD-like MF from true SD include unilateral presentation, asymmetry, and poor response to standard SD therapies. As such, we propose a set of evaluations to consider when managing a patient with atypical SD or MF plus a new SD-like rash: (1) full body skin examination for concurrent MF lesions, (2) shave biopsy and/or repeat biopsies for histologic/immunohistologic analysis, and (3) molecular testing for T-cell clonality.

## Conflicts of interest

None disclosed.
